# Sirtuin 1 as an emerging exerkine in the aging process: unveiling its multifaceted biological roles

**DOI:** 10.1007/s10522-026-10442-z

**Published:** 2026-04-28

**Authors:** Patrício Lopes de Araújo Leite, Larissa Alves Maciel, Rita Cristine Barboza Patricio, Geovanna Lopes Carneiro Pereira, Antônio Sérgio de Oliveira Lamounier, Samuel da Silva Aguiar, Caio Victor Sousa, Ivo Vieira de Sousa Neto, Herbert Gustavo Simões

**Affiliations:** 1 Graduate Program in Physical Activity, Health, and Human Performance, Catholic University of Brasilia (UCB), EPTC QS 7 LT 1, Bloco G, Sala G116, Taguatinga, DF 72.022-900 Brazil; 2 Graduate Program in Physical Therapy, University of Brasília (UNB), Ceilândia, Brazil; 3 Graduate Program in Medicine, Catholic University of Brasilia (UCB), Taguatinga, DF Brazil; 4 Department of Health and Human Sciences, Loyola Marymount University, Los Angeles, USA; 5 School of Physical Education and Sport of Ribeirão Preto, University of São Paulo (USP), Ribeirão Preto, São Paulo Brazil; 6 Graduate Program in Genomic Sciences and Biotechnology, Catholic University of Brasília, Brasília, Brazil

**Keywords:** Senescence, Sirtuins, Physical exercise, Aging

## Abstract

Sirtuin 1 (SIRT1) was initially identified as an enzyme that deacetylates histones and suppresses gene activity. Since then, its roles have expanded considerably, and it is now recognized as a multifunctional protein conserved across various organisms. Despite increasing interest, it remains essential to clarify how exercise-induced changes in SIRT1 counteract multiple hallmarks of aging, as well as the full scope of SIRT1’s impact on different physiological systems. This review highlights recent findings on the short- and long-term effects of exercise on SIRT1 signaling in both rodents and humans during aging. We explore the molecular pathways activated in various tissues, providing insight into the specific biological functions of SIRT1 within aging cells. Optimal levels of SIRT1 help maintain homeostasis and a biochemical environment conducive to healthspan, influencing biological processes such as mitochondrial dynamics, metabolic pathways, tissue remodeling, autophagy, inflammatory responses, and redox balance. This indicates that SIRT1, a pleiotropic molecule, orchestrates multiple responses throughout aging. SIRT1 may act as a dynamic sensor for exercise benefits and protect against aging by maintaining genomic integrity. Different exercise protocols (acute and chronic) and modalities (aerobic, resistance, and combined training) can increase mRNA levels, activity, or protein levels of SIRT1 in various vital organs (adipose tissue, hippocampus, heart, liver, bone, and skeletal muscle) of aged animals and older adults, promoting health. Taken together, these observations support the notion that SIRT1 functions as a potential exerkine, and understanding its role in exercise-induced adaptations offers new insights into non-pharmacological strategies to enhance longevity.

## Introduction

Aging is a complex and inevitable biological process characterized by progressive physiological, molecular, and cellular deterioration. Numerous anti-aging therapies have been proposed (Li et al. 2021), among which, physical exercise stands out as a particularly effective intervention due to its affordability and broad applicability across various organs and systems (Boström et al. 2013; Heinonen et al. 2014) exercise plays a critical role in extending healthspan and improving quality of life (Garatachea et al. 2015). Beyond clinical health, it also mitigates the major hallmarks of aging (Botella et al. 2024; Garatachea et al. 2015; Gleeson et al. 2011; Monda et al. 2017). Combined with a nutritionally balanced diet, exercise training can be considered a form of medicine for the aging population and should be prioritized to prevent chronic diseases and comorbidities.

With advancing aging, there is a decline in nicotinamide adenine dinucleotide (NAD^+^) availability and an increase in reactive oxygen species (ROS) with advanced age, leading to reduced transcriptional and translational levels of Sirtuin 1 (SIRT1), a member of the deacetylase family. Moreover, decreased SIRT1 during aging has been attributed to enhanced degradation via ubiquitination and autophagy. SIRT1 deacetylates target proteins and can either activate or inhibit molecules depending on the cellular context. One recently identified mechanism through which physical exercise exerts its positive effects on aging is through SIRT1 activation (Juan et al. 2023). Exercise orchestrates responses to metabolic stress and energy demands, leading to AMP-activated protein kinase (AMPK) phosphorylation, which in turn activates SIRT1. In addition, mechanical stimuli directly increase SIRT1 transcription, potentially via Forkhead box O 3 (FOXO3) release from the SIRT1 promoter (Pardo and Boriek 2011). Consequently, exercise-induced SIRT1 upregulation reduces oxidative stress, inflammation, apoptosis, and metabolic dysfunction in aged individuals, while enhancing mitochondrial biogenesis, primary metabolism, tissue remodeling and autophagy. Hence, the possibility that elevated SIRT1 activity may counteract age-related declines such as neurodegeneration, cardiovascular changes and metabolic disorders has gained increasing attention (Corrêa et al. 2022).

Despite extensive research on SIRT1 in aging, its pleiotropic responses remain insufficiently explored. In this context, it is essential to clarify how exercise-induced SIRT1 modulation counteracts multiple hallmarks of aging. While several studies have summarized the beneficial effects of exercise on SIRT1, most focus on specific diseases or single organs, or address adult rather than aged conditions (Juan et al. 2023). Synthesizing recent findings on how exercise regulates SIRT1 may thus provide new insights into the biological significance of aging and the role of SIRT1 as an exerkine. The SIRT1 signature could also highlight potential pharmacological and non-pharmacological targets, emphasizing the positive effects of exercise interventions on longevity.

This review provides a comprehensive overview of SIRT1 biological roles in aged organisms. We also clarify the acute and chronic impact of different exercise modalities on the SIRT1 signaling pathways in rodents and humans during the aging process, understanding the complete picture of SIRT1 in response to physical exercise. We further explore the molecular landscape across physiological systems in both sexes and propose the hypothesis that the long-term effects of SIRT1 adaptations to exercise may reflect coordinated demands across multiple tissues to support longevity. Finally, we summarize the main mechanisms of SIRT1 with potential therapeutic and clinical applications, offering insights and recommendations relevant to both basic and clinical research. Given the significant increase in the global aging population, these observations are relevant to the fields of biogerontology and exercise physiology.

## Multifaceted biological roles of SIRT1 during the aging process

### SIRT1 delays cellular senescence and stem cell exhaustion

The biological aging process is characterized by the progressive accumulation of damage that impairs molecular, cellular, and ultimately physiological functions, leading to an irreversible decline in systemic performance (Guo et al. 2022). This process is linked to a range of chronic diseases, including type 2 diabetes, cancer, neurodegenerative disorders and cardiovascular diseases (Chen et al. 2020), and is largely attributed to cellular senescence, a state in which cell proliferation, differentiation, and other vital functions are disrupted (Guo et al. 2022).

The identification of aging biomarkers is essential for understanding the underlying causes of aging and developing strategies aimed at mitigating its effects. Several hallmarks of aging have been proposed, with the article by López-Ótin et al. 2013 identifying nine hallmarks: stem cell exhaustion, altered intercellular communication, genomic instability, telomere attrition, epigenetic alterations, loss of proteostasis, dysregulated nutrient sensing, mitochondrial dysfunction, and cellular senescence (López-Otín et al. 2013). A update by López-Ótin et al. 2023 expanded this framework to include impaired autophagy, dysbiosis, and chronic inflammation to the list of aging hallmarks (López-Otín et al. 2023). Additionally, hormonal imbalances have been recognized as important contributors to the aging process (Pataky et al. 2021).

Over the decades, several approaches have been developed to counteract the effects of aging. Research has focused on enhancing nicotinamide adenine dinucleotide (NAD) concentration, a coenzyme present in its oxidized (NAD⁺) and reduced form (NADH). NAD^+^ plays a vital role in numerous enzymatic reactions, and its decline with age has been implicated in longevity and quality of life (Chini et al. 2024). NAD^+^ is tightly linked to the activity of sirtuins, a family of seven enzymes (SIRT1-SIRT7), with SIRT1 being the most extensively studied. SIRT1 is predominantly localized in the nucleus but can also translocate to the cytoplasm depending on the cellular context, cell type, metabolic state, and external stimuli. It is widely expressed across multiple tissues and organs, including skeletal muscle, adipose tissue, heart, brain, kidneys, and lungs. (Lee et al. 2019). As a class III histone deacetylase, SIRT1 requires NAD + to exert its enzymatic function, during which NAD⁺ is cleaved into nicotinamide (NAM) and O-acetyl-ADP-ribose (Wu et al. 2022).

SIRT1 is intimately involved in metabolic regulation and aging, exerting protective effects against a wide range of age-associated conditions, including neurodegenerative diseases, cancer, cardiovascular diseases, increased adiposity, insulin resistance, pancreatic beta-cell impairment, glucose homeostasis dysregulation, and hepatic steatosis (Wu et al. 2022; I. H. Lee 2019). Although its direct role in extending human lifespan remains inconclusive, the age-associated decline in SIRT1 expression has been linked to diminished mitotic capacity and replicative senescence through downregulation of the p53/p21 pathway (Xu et al. 2020).

Mesenchymal stem cell-derived exosomes (MSC-Exos) exert important immunomodulatory effects due to the self-renewal and differentiation capacity of their parent cells. In senescent SAMP8 mice, SIRT1 expression was markedly reduced, whereas MSC-Exos significantly restored its expression. In parallel, MSC-Exos attenuated oxidative stress and apoptosis in neural tissue, thereby delaying brain aging. These findings indicate that SIRT1 reactivation may be a key mechanism underlying the neuroprotective effects of MSC-Exos. Given the central role of SIRT1 in redox homeostasis, apoptosis regulation, and neuronal survival, these findings support the hypothesis that reducing oxidative stress and apoptosis through SIRT1 signaling may represent a promising strategy for the prevention and treatment of age-related neurodegenerative disorders (Zhang et al. 2023).

Furthermore, bone marrow-derived MSCs isolated from aged rats exhibited lower SIRT1 mRNA expression, reduced protein levels, and decreased enzymatic activity compared with MSCs derived from young rats. Functionally, SIRT1 silencing in young rat MSCs induced cellular senescence, reduced proliferative capacity, and increased DNA damage, whereas SIRT1 overexpression in aged rat MSCs reversed senescence-related phenotypes and improved proliferation. Collectively, these findings suggest that SIRT1 plays a central role in preserving the regenerative potential of MSCs during aging by attenuating senescence and enhancing proliferation (Chen et al. 2014).

Recent evidence has demonstrated that soluble α-Klotho (sKL) supplementation may enhance proliferative capacity in cardiomyocytes, renal tubular epithelial cells, and progeroid human fibroblasts through SIRT1-dependent mechanisms. One of the main pathways involved appears to be the Klotho–SIRT1–Checkpoint kinase 2 (CHK2) axis, in which CHK2, a central DNA damage checkpoint kinase, inhibits cell cycle progression in response to genomic instability. In aged mice, SIRT1 expression was markedly reduced in multiple organs, whereas 10 weeks of sKL treatment restored SIRT1 levels in both aged wild-type and Klotho-deficient mice. Mechanistically, SIRT1 was required for the inhibitory effect of sKL on CHK2 activity and for the proliferative response observed in cardiomyocytes, renal tubular cells, and progeroid fibroblasts. Since Ataxia-telangiectasia mutated kinase (ATM) recruits SIRT1 to sites of DNA damage, SIRT1 may act as an important regulator of CHK2 activity and cell cycle progression. By restoring SIRT1 expression, sKL attenuated DNA damage signaling, reduced CHK2 overactivation, and increased the expression of cell cycle marker (Dai et al. 2025).

Concomitantly, evidence indicates that SIRT1 is a central regulator of organismal lifespan, preserving stem cell functions. Under CR, SIRT1 modulates cellular differentiation of stem cells by counteracting adipocyte switching, a process in which adipocyte formation increases at the expense of osteoblast generation in the bone marrow. CR promotes osteogenic differentiation through SIRT1-mediated mechanisms (Chen et al. 2024). Molecularly, SIRT1 can silence genes involved in cell differentiation, thereby maintaining the undifferentiated state of stem cells and its capacity for self-renewal, besides influencing the transcription of genes that control stem cell identity and function, ensuring multipotency (Chen et al. 2024). Thus, the role of SIRT1 in maintaining stem cell stemness can prevent apoptosis and modulate cellular senescence, ultimately contributing to tissue regeneration and potentially extending life expectancy.

### SIRT1 contributes to telomere maintenance

Stem cell depletion during aging has been closely associated with telomere shortening. Telomeres are nucleoprotein structures composed of tandem TTAGGG repeats that cap the ends of eukaryotic chromosomes (Blackburn 1991). Their primary role is to maintain chromosome integrity and stability. With each cell division, telomeres progressively shorten, contributing to genomic instability and increasing the risk of age-related diseases such as coronary artery disease, vascular disease, Alzheimer’s, cancer, and cellular senescence (Blackburn et al. 2015).

SIRT1 contributes to telomere preservation by regulating histone deacetylation and chromatin remodeling (Amano and Sahin 2019). In mouse embryonic fibroblasts, SIRT1 overexpression is associated with elongated telomeres, whereas SIRT1 deficiency results in telomere shortening. In aging models, elevated SIRT1 expression have also been linked to improved telomere maintenance (Lee et al. 2024; Palacios et al. 2010).

In telomerase reverse transcriptase (TERT) knockout mice, telomere dysfunction reduces hepatic SIRT1 concentration. Dysfunctional telomeres promote sustained p53 activation, which induces miR-34a, thereby suppressing SIRT1 expression through direct binding to the 3′UTR region of SIRT1 mRNA (Amano et al. 2019).

Moreover, SIRT1 upregulation reduced γ-H2AX foci and telomere dysfunction-induced foci (TIFs), suggesting a protective role against age-related DNA damage through modulation of telomere function. Mechanistically, SIRT1 promoted TERT expression, enhanced telomerase activity, and positively regulated tripeptidyl peptidase 1 (TPP1), a shelterin complex component involved in telomere protection. These findings suggest that SIRT1 may preserve telomere integrity and genomic stability during aging through telomere-related mechanisms (Chen et al. 2014).

Humans with long-standing type 1 diabetes mellitus (T1DM) reported both reduced SIRT1 expression and shorter telomere length compared with age-matched controls (Opstad et al. 2021). Furthermore, Pieters et al. (2015) reported that SIRT1 expression partially mediates approximately 40% of the association between telomere length and mitochondrial DNA (mtDNA) content in older adults (Pieters et al. 2015). In the exercise context, master athletes demonstrated a positive relationship between higher SIRT1 concentrations and longer telomere length, suggesting an important role of exercise in improved telomere maintenance (Aguiar et al. 2022).

### SIRT1 as a critical target that modulates DNA repair and genomic instability

DNA damage is closely associated with cellular aging and often arises from oxidative stress, contributing to genomic instability. Examples include base modifications and single- or double-strand breaks, which can cause severe and often irreparable genomic damage (Alves-Fernandes and Jasiulionis 2019). Despite these threats, cells possess sophisticated mechanisms to detect, prevent, and repair DNA damage, processes that are vital for maintaining genomic integrity, organismal homeostasis, and cell survival (Lagunas-Rangel 2019).

SIRT1 influences genome architecture by deacetylating heterochromatin regions and extrachromosomal rDNA circles (ERCs), thereby contributing to nucleolar disassembly, DNA regulation, and repair (Alves-Fernandes and Jasiulionis 2019). Under stress conditions, SIRT1 localizes to sites of DNA damage and modulates histone deacetylation, facilitating chromatin reorganization and transcriptional silencing (Alves-Fernandes and Jasiulionis 2019). In addition, SIRT1 targets several critical DNA repair proteins (Alves-Fernandes and Jasiulionis 2019), including poly (ADP-ribose) polymerase 1 (PARP1), Ku heterodimer 70 (Ku70), Nijmegen Breakage Syndrome (NBS) protein, and Werner syndrome helicase (WRN), xeroderma pigmentosum group A (XPA), p53, ATM, and CHK2 (Alves-Fernandes and Jasiulionis 2019; Lee et al. 2019). Through these interactions, SIRT1 enhances the efficiency of multiple DNA repair pathways, including base excision repair (BER), nucleotide excision repair (NER), mismatch repair (MMR), non-homologous end joining (NHEJ), and homologous recombination repair (HR) (Alves-Fernandes and Jasiulionis 2019; Lagunas-Rangel 2019).

In MMR, SIRT1 regulates the stability and activity of MSH2 and MSH3. During double-strand break repair, SIRT1 deacetylates NBS1, Ku70, Human Males Absent on the First (hMOF), and WRN, thereby enhancing DNA repair capacity while also promoting their degradation to prevent potentially deleterious activities. Moreover, the interaction between SIRT1 and WRN regulates its helicase activity and facilitates WRN reentry into the nucleolus, as excess WRN is targeted for degradation (Lagunas-Rangel 2019). SIRT1 also interacts with ATM and CHK2, two major checkpoint regulators of the DNA damage response, reinforcing its role as a key mediator of genomic stability and cell survival (Kwon et al. 2019).

### SIRT1 alleviates proteostatic stress involving the rapamycin signaling pathway and autophagy process

Proteostasis represents an intricate network of pathways that govern protein synthesis, folding, trafficking, and degradation. Damaged proteins must be either refolded or degraded, processes mediated primarily by molecular chaperones and the ubiquitin–proteasome system. SIRT1 has been reported to positively modulate protein quality, as Sirt1 deficiency induces the accumulation of ubiquitinated proteins in cells and tissues, especially under heat stress. Furthermore, downregulation of SIRT1 decreased basal expression of Hsp70, impairing protein survival (Tomita et al. 2015). At the single cell level, SIRT1-deficient cells exhibit a higher protein content per cell when compared to wild-type, a process explained by SIRT1 interacting with TSC2, a component of the mTOR inhibitory complex (Latorre et al. 2024).

Autophagy is a highly conserved catabolic process essential for maintaining proteostasis and cellular homeostasis, especially under conditions of metabolic stress. This mechanism selectively degrades and recycles nuclear and cytoplasmic components, lipids, and dysfunctional proteins, to support cellular renewal and survival. Autophagy is tightly regulated by key signaling molecules, including mTORC1, AMPK, protein kinase B (AKT), and SIRT1 (Nakatogawa 2020).

Emerging evidence indicates that SIRT1 contributes to autophagy regulation at multiple stages, beginning with its initiation. During this phase, SIRT1 stabilizes tuberous sclerosis complex 2 (TSC2), which negatively regulates the mTORC1 pathway by inactivating the brain-enriched Ras homologue (RHEB) (Kim et al. 2022). RHEB activates mTORC1, whereas the GTPase-activating protein (GAP) activity of TSC2 enhances GTP hydrolysis. Increased TSC2 stability enhances GTP hydrolysis, thereby downregulating the mTORC1 signaling process and initiating the autophagy process (Hoseini et al. 2025).

SIRT1 also promotes autophagy initiation through the deacetylation of Unc-51-like (ULK1) and Unc-51 Like Autophagy Activating Kinase 2 (ULK2), which are essential for the autophagy cascade. At the molecular level, SIRT1-mediated deacetylation enhances ULK1/ULK2 activity under stress conditions, promoting the assembly of the autophagy initiation complex and the degradation of dysfunctional organelles (Ge et al. 2022).

Under hypoxic conditions, SIRT1 regulates autophagy through the Bcl-2/adenovirus E1B 19 kDa-interacting protein 3 (BNIP-3) pathways. SIRT1-mediated deacetylation of FOXO3 enhances its transcriptional activity, enabling FOXO3 to bind to the BNIP3 promoter and induce its expression. BNIP3 disrupts the Bcl-2–Beclin1 complex, enabling Beclin1 to activate the class III phosphatidylinositol 3-kinase (PI3K) complex and initiate autophagy (Kim et al. 2022). In fluoride-treated MC3T3-E1 osteoblasts, increased SIRT1 expression protects against apoptosis by enhancing FOXO3 deacetylation and BNIP3-mediated autophagic flux (Ding et al. 2024).

During the elongation and maturation phase, SIRT1 facilitates the formation of the ATG16L1-ATG5-ATG12 complex via the deacetylation of ATG5, ATG7 and ATG12, thereby promoting the autophagosome membrane expansion. In addition, SIRT1 regulates the translocation of nuclear microtubule-associated protein 1A/1B-light chain 3 (LC3-I), enabling its conversion to LC3-II and insertion into the autophagosomal membrane (Kim et al. 2022).

Furthermore, mitophagy appears to be mediated, at least in part, by the SIRT1/SIRT3–FOXO1/3 axis. In mice, increased SIRT3 expression deacetylated and activated FOXO3A. The activated FOXO3A, in turn, upregulated Parkin expression, favoring its recruitment to damaged mitochondria and promoting mitophagy (Yu et al. 2017).

In parallel, exercise reduced mitochondrial PINK1 and P62 accumulation and increased Parkin recruitment in mice, indicating improved mitophagic flux. Since PINK1 accumulates in damaged mitochondria and recruits Parkin to initiate their clearance, these findings suggest that exercise enhanced the efficiency of PINK1/Parkin-mediated mitophagy rather than simply increasing mitochondrial damage signals (Zhao et al. 2023).

### SIRT1 integrates primary metabolism, modifying mitochondrial biogenesis and function

Through the deacetylation of PGC-1α, SIRT1 integrates primary metabolism by upregulating genes involved in mitochondrial function (ERRα), the tricarboxylic acid cycle (TCA), electron transport chain components (cytochrome c and COXVa), fatty acid metabolism (MCAD), and energy regulation (PDK4), while reinforcing its expression. This regulatory network becomes particularly important under conditions of increased energy demand or caloric restriction (CR), where SIRT1 activation is facilitated by AMPK. Elevated Adenosine monophosphate (AMP) concentration during caloric restriction (CR) and physical exercise activates AMPK, which subsequently enhances lipid metabolism to meet cellular energy requirements. In addition, AMPK upregulates nicotinamide phosphoribosyl transferase (NAMPT) expression, thereby stimulating NAD^+^ biosynthesis and amplifying SIRT1 activity (Chen et al. 2025).

The upregulation of SIRT1 within the AMPK pathway has been linked to improved systemic insulin sensitivity (Wu et al. 2022). In the skeletal muscle, activation of the AMPK-SIRT1 signaling axis promotes fatty acid utilization and suppresses glycolytic flux, contributing to greater metabolic flexibility. In mice with moderate SIRT1 overexpression, enhanced glucose tolerance and insulin sensitivity have been observed even under a low-fat diet. These metabolic improvements are associated with increased brown adipose tissue (BAT) activity, likely mediated by an amplified response to adrenergic stimulation (Chen et al. 2025).

SIRT1 functions as a cellular energy sensor, activated by fluctuations in intracellular NAD⁺ levels. Mitochondria, the central organelles responsible for adenosine triphosphate (ATP) synthesis, oxidize carbohydrates and fatty acids to generate ATP, the universal energy currency that sustains cellular processes (Spinelli and Haigis 2018). Beyond ATP production, mitochondria regulate additional critical functions, including ROS generation and detoxification of ROS, apoptosis, calcium homeostasis, metabolic synthesis and degradation, and organelle dynamics. Disruptions in these processes contribute to mitochondrial dysfunction, which has been implicated in the pathogenesis of numerous disorders, including neurodegenerative diseases, myopathies, and type 2 diabetes (Wang et al. 2025). SIRT1 protects against mitochondrial dysfunction primarily through the deacetylation of FOXO transcription factors, particularly under stress conditions. By modulating FOXO activity, SIRT1 influences nuclear gene expression that governs mitochondrial function and facilitates retrograde signaling between mitochondria and the nucleus (Luen Tang 2016; Xu et al. 2023).

By deacetylating and activating FOXO1 and FOXO3, SIRT1 induces PGC-1α expression, a master regulator of mitochondrial biogenesis that coordinates the transcription of genes involved in oxidative phosphorylation and fatty acid oxidation. PGC-1α activation enhances mitochondrial respiratory capacity and energy efficiency, initiating a transcriptional cascade that supports metabolic adaptation (Guan et al. 2025). For example, nuclear respiratory factor 1 (NRF-1), co-activated by PGC-1α under SIRT1 regulation, governs the expression of nuclear genes that encode mitochondrial proteins (Fernandez-Marcos and Auwerx 2011). NRF1 specifically controls genes required for mitochondrial DNA replication and transcription, as well as the assembly of respiratory chain complexes. A key downstream target of NRF1 is mitochondrial transcription factor A (TFAM) (Scarpulla 2006), which binds mtDNA to stabilize its structure and regulate the transcription of genes required for oxidative phosphorylation and ATP synthesis (Chen et al. 2025; Lee et al. 2024; Scarpulla 2008).

**Fig. 1:**
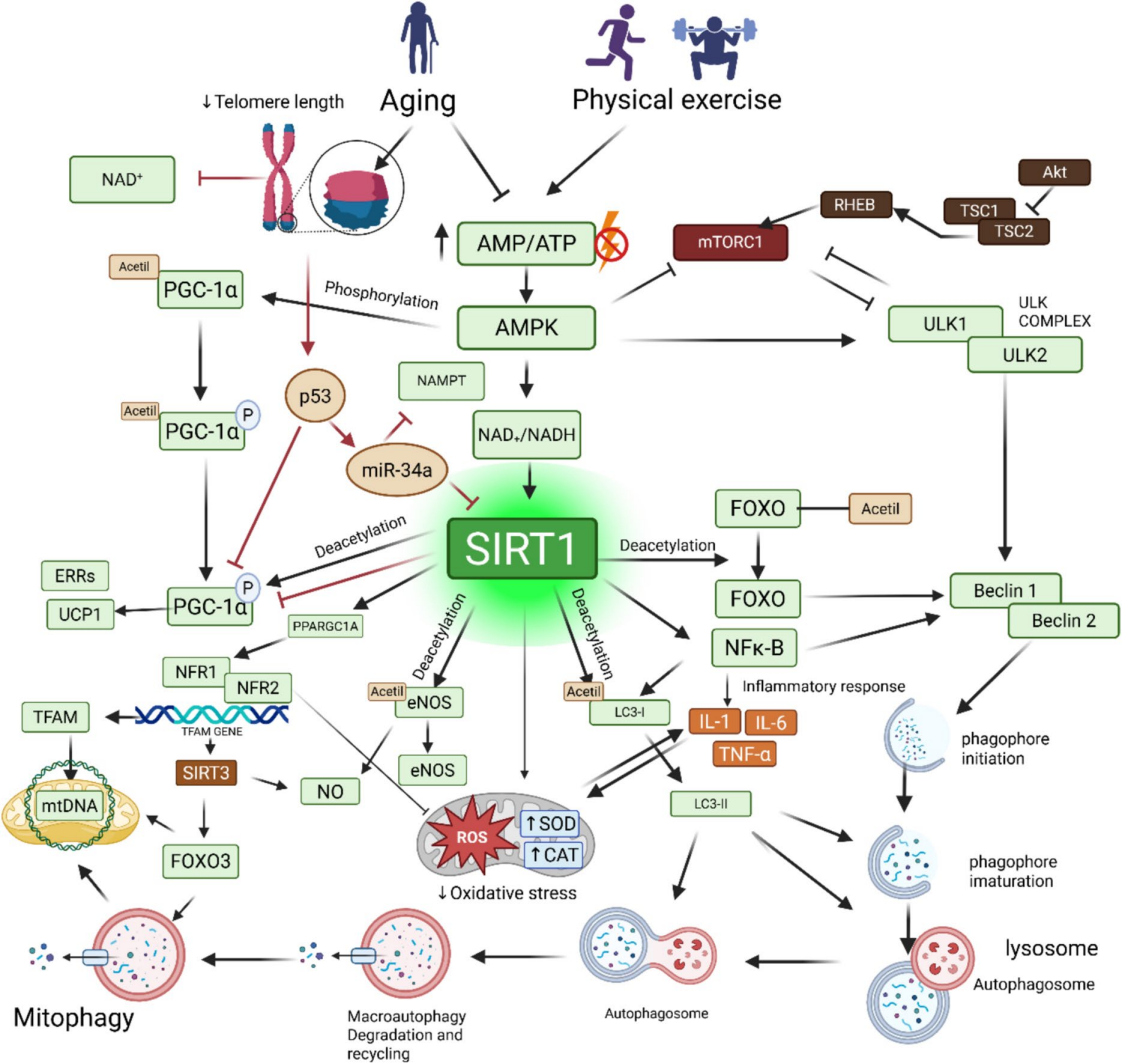
Aging and exercise-induced opposition effects on AMPK activation and its subsequent stimulation of SIRT1 activity. Aging decreases telomere length and NAD⁺ availability, while increasing ubiquitination and proteasomal degradation. In contrast, exercise enhances the AMP/ATP ratio, thereby activating AMPK, SIRT1, and the PGC1-α axis, which promotes an increase in NAD⁺ availability and NRF1 and NRF-2 activation. Subsequently, there is an increase in uncoupling proteins, mitochondrial respiratory chain and antioxidant capacity by upregulation of SOD and catalase. Notably, a positive feedback loop also exists, whereby activated SIRT1 enhances AMPK signaling through deacetylation of LKB1, further supporting cellular energy homeostasis and metabolic resilience that reduces chronic inflammation, decreasing TNF-α, IL-1β, and IL-6. Also, SIRT1 alleviates proteostatic stress involving the rapamycin signaling pathway and the autophagy process by the ULK complex

Under nutrient deprivation, SIRT1 promotes gluconeogenesis through the deacetylation of PGC-1α and FOXO, thereby upregulating gluconeogenic genes, enhancing fatty acid oxidation, and suppressing both lipogenesis and glycolysis. The inhibition of glycolysis is mediated in part by SIRT1-induced repression of hypoxia-inducible factor 1α (HIF-1α) and by PGC-1α-dependent regulation of glycolytic genes, as well as through the deacetylation of phosphoglycerate mutase 1 (PGAM1). Collectively, these actions redirect metabolism toward oxidative pathways during fasting and caloric restriction (Zhang et al. 2021; Ye et al. 2017). Figure 1 provides an overview of the molecular pathways mediating SIRT1 signaling during aging and exercise training.

### SIRT1 regulates dysbiosis dynamics and gut health

The intestinal microbiota is highly dynamic and shaped by dietary and environmental factors. When its functional and compositional balance is disrupted beyond resilience threshold, a state of dysbiosis arises. Dysbiosis has been associated with a range of multifactorial diseases and can be categorized into three non-mutually exclusive types: loss of beneficial microorganisms, expansion of potentially pathogenic species, and a reduction in overall microbial diversity (Shang et al. 2024).

SIRT1 plays a critical role in maintaining intestinal epithelial homeostasis by regulating inflammation and oxidative stress. SIRT1 suppresses Paneth cell function and increases gut epithelial sensitivity to environmental stress. In mice, deletion of SIRT1 in Paneth cells elevates Wnt signaling and ATF4/endoplasmic reticulum stress pathways, leading to increased Paneth cell abundance and antimicrobial peptide production, improved protection against intestinal immune cell expansion and greater resistance to chemically induced colitis (Garcia-Peterson et al. 2026).

Resveratrol enhances SIRT1 activity, strengthening its anti-inflammatory and antioxidant effects (Yu et al. 2019). Resveratrol attenuates colitis by reversing colitis-induced microbial dysbiosis. The resulting microbiota profile helps protect against colonic inflammation by increasing regulatory T cells (Tregs) and suppressing inflammatory Th1 and Th17 responses (Alrafas et al. 2019). Moreover, SIRT1 deficiency and intestinal dysbiosis may also affect systemic organs. Evidence indicates a connection between gut dysbiosis and kidney dysfunction: in cirrhotic mice, intestinal SIRT1 expression mitigated TNFα-mediated oxidative stress and protected against renal injury (Chou et al. 2021).

The central nervous system (CNS) is likewise influenced by intestinal dysbiosis. Altered microbial communities have been implicated in neuronal protein aggregation, a hallmark of neurodegenerative diseases. In Parkinson’s disease, for instance, gram-negative bacteria produce abundant lipopolysaccharides (LPS), which promote α-synuclein aggregation and neuronal death. Given SIRT1’s established role in anti-inflammatory signaling and intestinal barrier regulation, intestinal SIRT1 likely contributes to microbial homeostasis and CNS protection (Chandramowlishwaran et al. 2020). Furthermore, mice treated with Lactobacillus rhamnosus GG exhibited reduced inflammation through activation of the SIRT1/AMPK/PGC-1α pathway, leading to decreased NF-κB expression, a master regulator of inflammation (Liu et al. 2021).

### SIRT1 alleviates oxidative stress and inflammation in aging

SIRT1 regulates endothelial nitric oxide synthase (eNOS) activity by directly deacetylating the enzyme, thereby enhancing nitric oxide (NO) production. Endothelial SIRT1 plays an important vasoprotective role by regulating several proteins, including endothelial nitric oxide synthase (eNOS). In endothelial cells, SIRT1 and eNOS act synergistically through positive feedback mechanisms that help maintain endothelial function. Recent evidence also indicates that SIRT1 contributes to the regulation of perivascular adipose tissue (PVAT) function, arterial remodeling, and vascular aging (Man et al. 2019).

Oxidative stress arises from an imbalance between free radical production and antioxidant defenses. Endogenous antioxidant enzymes, such as superoxide dismutase (SOD) and catalase (CAT), are essential for cellular protection (Córdova et al. 2000). Under oxidative stress conditions, AMPK acts as an energy sensor and activates SIRT1, which subsequently stimulates the FOXO transcription factor. This cascade contributes to improved mitochondrial function, reduced mitochondrial damage, and attenuation of oxidative stress-induced cellular injury (Guan et al. 2025). Additionally, SIRT1 deacetylates NRF-2, facilitating its translocation to the nucleus and increasing its transcriptional activity, which further amplifies the antioxidant response. SIRT1 also influences p53 by deacetylating it. SIRT1-mediated deacetylation of p53 supports DNA repair and cell survival. (Lao et al. 2025; Ong and Ramasamy 2018).

SIRT1 is involved in deacetylating enzymes associated with oxidative stress and plays a role in attenuating inflammatory processes through various signaling pathways. For instance, SIRT1 promotes the deacetylation of NF-κB subunits, thereby inhibiting NF-κB activity and reducing pro-inflammatory cytokines (TNF-α, IL-1β). This inhibition occurs because SIRT1 negatively regulates P65 acetylation by deacetylating it at lysine 310, resulting in anti-inflammatory effects (Yang et al. 2010). Additionally, the acetylation of P65 at lysine 310 influences the methylation of lysines, which accelerates the ubiquitination and subsequent degradation of P65 (Lei et al. 2012). Beyond its effects on NF-κB signaling, SIRT1 may also regulate inflammatory macrophage polarization and oxidative stress through other pathways. Experimental evidence demonstrated that SIRT1 attenuated M1 macrophage polarization by suppressing MAPK/NF-κB/AP-1 signaling while simultaneously activating the antioxidant Nrf2/HO-1 pathway (Zhao et al. 2024). Furthermore, deletion of SIRT1 in hepatocytes caused liver fibrosis and a pro-inflammatory response via NLRP3 and IL‐1β induction in young mice, mimicking the effects of aging-induced inflammation and tissue fibrosis (Adjei‐Mosi et al. 2023).

Additionally, SIRT1 participates in several pathways that regulate immunosenescence. In mice, microglial SIRT1 appears to play an important role in protecting the aging brain against neuroinflammation and cognitive decline. Reduced SIRT1 expression in microglia has been associated with increased IL-1β production, exacerbated microglial activation, enhanced tau-related pathology, and worse memory performance (Cho et al. 2015).

The SIRT1–FoxO1 axis appears to play an important role in human immunosenescence, particularly in the accumulation of CD8 + CD28 − T cells. These cells show reduced SIRT1 levels, which is associated with greater metabolic activation even at rest. FoxO1 expression also declines in parallel with SIRT1, although its mRNA levels remain unchanged, suggesting post-transcriptional regulation. In the absence of SIRT1, FoxO1 is degraded by the proteasome. Overall, the progressive reduction in SIRT1 and FoxO1 with T-cell differentiation may contribute to the pro-inflammatory and dysfunctional phenotype observed in aging CD8 + T cells (Jeng et al. 2018).

### Significant role of SIRT1 in hormonal regulation

Steroid hormone receptors, including estrogen receptors (ER) and androgen receptors (AR), are members of the nuclear receptor family of eukaryotic transcription factors (Davey and Grossmann 2016). Their signaling is essential for maintaining hormonal homeostasis. Hormone-receptor complexes bind chromatin and alter its structure, thereby facilitating or restricting the recruitment of transcriptional co-regulators. Under conditions of overstimulation, these receptors can promote tumorigenic processes by driving cell proliferation and inhibiting apoptosis (Weidemüller et al. 2021).

In both in vivo and in vitro models, hyperactivation of steroid hormone receptors can result from post-translational modifications, particularly acetylation mediated by histone acetyltransferases (HATs) (Wu et al. 2022). This mechanism is particularly relevant in oncogenesis, as deregulated acetylation disrupts endocrine signaling and contributes to tumor initiation and progression (Siblini et al. 2022). SIRT1 can coactivate steroid hormone receptors, such as ERα, even in the absence of ligand binding (Bayele 2020). Moreover, in Leydig cells, SIRT1 plays a central role in testosterone biosynthesis by modulating autophagy and enhancing cholesterol uptake, a rate-limiting step in steroidogenesis (Bayele 2020; Khawar et al. 2021).

In this context, SIRT1 also regulates the hypothalamic–pituitary axis (Yamamoto and Takahashi 2018). Although human evidence remains limited, physically active individuals exhibit higher SIRT1 and testosterone levels than sedentary controls, suggesting that regular exercise may attenuate age-related declines in androgen production (Leite et al. 2024).

**Fig. 2:**
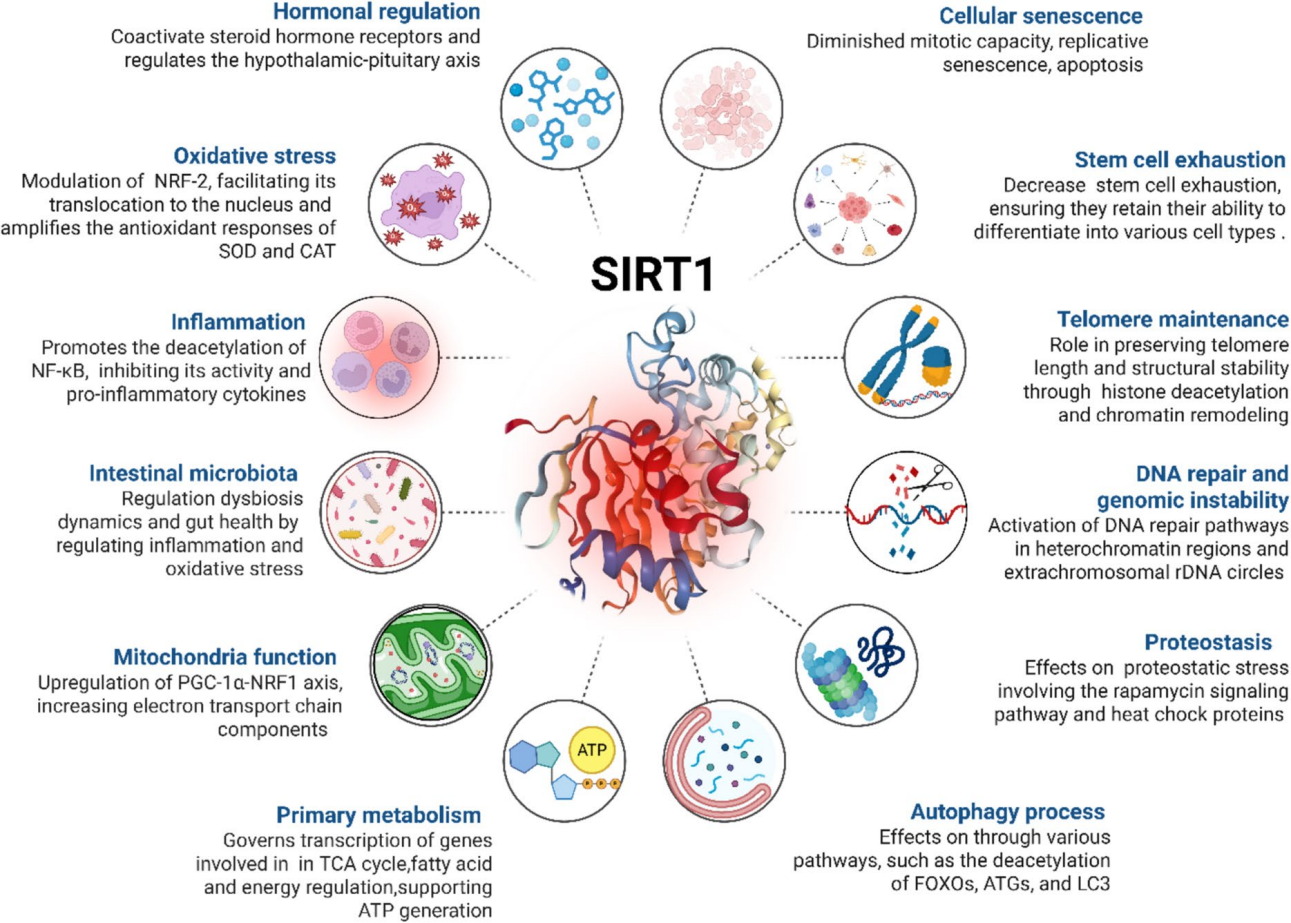
The main biological processes regulated by SIRT1 during the aging process. Multifaceted roles including cellular senescence, stem cell exhaustion, telomere maintenance, DNA repair and stability, proteostasis, autophagy process, primary metabolism, mitochondria function and dynamics, intestinal microbiota, inflammation, oxidative stress, and hormonal regulation. Therefore, SIRT1 plays a central role as a stress-responsive sensor that significantly impacts vital cellular responses. Biorender® web-based software was used to create the figure

Figure 2 summarizes the pleiotropic actions and multifaceted biological roles of SIRT1 during the aging process. Consequently, increasing SIRT1 counteracts multiple hallmarks of aging.

## Mechanisms of interaction between SIRT1 and physical exercise

### Multi-systemic actions of chronic exercise on SIRT1 levels in old animals

Chronic exercise protocols and various modalities increase SIRT1 activity or protein levels in adipose tissue, hippocampus, heart, liver, bone, and skeletal muscle in animal models, suggesting that this versatile molecule induces pleiotropic responses. Acting as an adaptive mechanism, upregulation of SIRT1 in multiple organs and tissues of aged animals contribute to improved mitochondrial metabolism, tissue remodeling, inflammation control, and redox balance.

Skeletal muscle progressively deteriorates with age due to reductions in mass and function, both of which are critical for tissue quality (Day et al. 2010). A pioneering study demonstrated that six weeks of treadmill exercise activated SIRT1 in the skeletal muscle of aged rats, primarily through its influence on nicotinamide phosphoribosyltransferase, which increased NAD⁺ synthesis, an essential molecule that supports SIRT1 activity. Exercise also helped restore the redox balance disrupted by aging, as trained animals showed markedly reduced protein carbonylation levels (Koltai et al. 2010). Ryall et al. (2015) investigated skeletal muscle regeneration following injury in both young and aged animals. They observed reduced satellite cell activity, impaired regenerative capacity, and diminished muscle strength in aged SIRT1 knockout mice compared with wild-type controls. Moreover, muscle contractile force after injury recovery was higher in aged mice overexpressing muscle-specific SIRT1 than in age-matched SIRT1 knockout animals. Aerobic exercise has also been shown to counteract sarcopenia by stimulating the PGC-1α–SIRT1 axis and lowering myostatin and FOXO3 expression in older rats (Neto et al. 2023). Collectively, these findings emphasize the role of SIRT1 in preserving muscle function and regenerative capacity during aging.

Osteoporosis is a common systemic condition associated with aging, characterized by reduced bone mass and mineral density, impaired microarchitecture, and increased fracture susceptibility. Exercise improves bone health during aging by promoting bone formation, in part through osteogenic differentiation of BMSCs via SIRT1-mediated autophagy (Zhu et al. 2023). In another study, old (12-month-old) and young (5-month-old) C57BL/6 J male mice underwent eight weeks of aerobic training. Greater SIRT1 expression was observed in both groups after training. Old trained mice also showed increased bone formation capacity and bone mass, as evidenced by greater trabecular volume, number, and thickness, reinforcing the positive effects of SIRT1 on bone homeostasis during aging (Zhu et al. 2023).

Aging negatively affects the liver and adipose tissue by inducing cellular senescence, inflammation, and mitochondrial dysfunction, collectively disrupting metabolic processes. Liver-specific SIRT1 knockout mice developed hepatic steatosis and premature senescence (Wang et al. 2010). In contrast, exercise increased SIRT1 expression in the liver of aged mice, restoring its levels and activating downstream signaling pathways. This adaptation reduced inflammation and lipid accumulation, promoted fatty acid oxidation, and helped reverse age-related hepatic dysfunction (Yang et al. 2024).

Similarly, adipose tissue, a metabolically active organ, is strongly regulated by mitochondrial activity and SIRT1. Thirupathi et al. (2019) examined young and old sedentary or trained rats subjected to strength or aerobic training. After eight weeks, trained groups exhibited reduced epididymal, retroperitoneal, and mesenteric adipose depots compared to sedentary controls. In parallel, PGC-1α, SIRT1, and pAMPK levels were higher in both trained groups than in old sedentary animals, confirming that exercise-induced mitochondrial biogenesis in adipose tissue is enhanced (Thirupathi et al. 2019).

Exercise-induced SIRT1 upregulation also benefits the cardiovascular system. In rats aged 3, 12, and 18 months subjected to 12 weeks of training, the AMPK–SIRT1–PGC-1α pathway was significantly higher in the hearts of trained aged animals compared to sedentary counterparts, suggesting an alternative cardioprotective mechanism (Lai et al. 2014). Chen et al. (2018) also studied the combined effects of aging and swimming exercise in rats with D-galactose-induced aging. They reported greater SIRT1 expression in the left ventricle of aged trained rats than in untrained ones, along with reduced TNF-α levels, thereby alleviating chronic inflammation (Chen et al. 2018).

Neuroprotective effects of exercise-induced SIRT1 activation have also been observed. In a study of D-galactose-induced hippocampal aging, 12 weeks of training increased AMPK, PGC-1α, and SIRT1 expression, while reducing TNF-α, NF-κB, cyclooxygenase-2 (COX-2), and inducible nitric oxide synthase (Lin et al. 2020). These changes attenuated aging-related inflammatory pathways, promoted neurogenesis, decreased oxidative stress, and enhanced resilience against neurodegeneration. By activating SIRT1, exercise helps counteract hippocampal decline and improves cognitive performance during aging (Mishra et al. 2021).

**Fig. 3:**
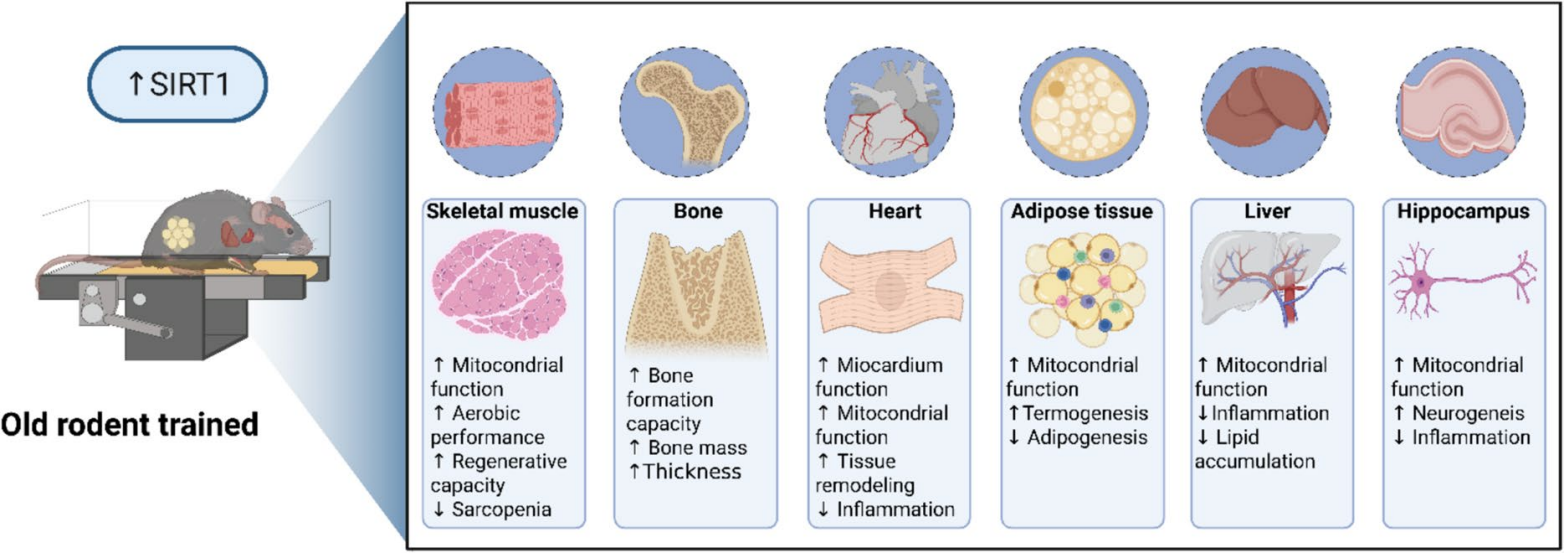
Multi-systemic actions of SIRT1 induced by physical exercise in the old animals. There is an upregulation of SIRT1 in skeletal muscle (biological process: ↑ mitochondrial function, aerobic performance, regenerative capacity, and ↓ sarcopenia), bone (biological process: ↑ formation capacity, bone mass, and thickness), heart (biological process: ↑ myocardium function, mitochondrial function, tissue remodeling, and ↓ inflammation), adipose tissue (biological process: ↑ mitochondrial function, thermogenesis, and ↓ adipogenesis), liver (biological process: ↑ mitochondrial function, ↓ inflammation and, ↓ lipid accumulation) and hippocampus (biological process: ↑ mitochondrial function, neurogenesis and ↓ inflammation). Biorender® web-based software was used to create the figure

Figure 3 illustrates the primary effects of SIRT1 induced by exercise training in the physiological systems of aged rodents.

### Effects of acute and chronic exercise on SIRT1 concentration in the older adults

In older adults, a single bout of exercise can induce a transient increase in SIRT1 activity in both skeletal muscle and circulation, primarily as a response to metabolic stress and the robust activation of cellular signaling pathways. This effect is likely mediated by AMPK, a key cellular energy sensor, during exercise (Bori et al. 2012). The first evidence regarding exercise-induced changes in human skeletal muscle SIRT1 expression was reported more than a decade ago. In a landmark study, a single session of acute aerobic exercise (45 min of treadmill running at 70–75% of VO_2_max until exhaustion) increased SIRT1 gene expression immediately after exercise in older sedentary individuals (Bori et al. 2012). This finding was corroborated by the same protocol, which enhanced SIRT1 expression in human skeletal muscle while reducing the expression of Fis1 and Mfn1 mRNA compared to sedentary older controls, thereby attenuating the deleterious effects of aging (Bori et al. 2012).

While acute exercise promotes only transient increases in SIRT1, regular exercise training (i.e., repeated sessions over time) produces more sustained and beneficial adaptations, including improvements in redox homeostasis, mitochondrial function, inflammation reduction, and enhanced cellular protection. Villanova et al. (2013) were among the first to explore the interaction between aging, exercise, and SIRT1 activity (Villanova et al. 2013). Their study demonstrated that SIRT1 activity in PBMCs declines after the age of 40, whereas it is upregulated in trained athletes.

Yang et al. conducted an elegant study investigating the effects of 16 weeks of progressive resistance training (RT) on SIRT1 mRNA expression in PBMCs of postmenopausal women with overweight/obesity. The intervention significantly upregulated SIRT1 expression, reduced Metabolic Dysfunction-Associated Steatotic Liver Disease (MASLD) biomarkers and risk indices, and decreased body fat mass (Yang et al. 2024). Similarly, 12 weeks of RT increased serum SIRT1 concentrations in older men, accompanied by elevations in PGC-1α and telomerase, thus suggesting potential anti-aging effects (Hooshmand-Moghadam et al. 2020). Additionally, a recent study in obese adults subjected to different oxygen conditions demonstrated that four weeks of exercise training in a normobaric hypoxia chamber upregulated SIRT1 expression, an effect absent under normoxic training or with remote ischemic preconditioning (Mozaffaritabar et al. 2024).

Evidence also indicates that SIRT1 contributes to cardiovascular health in older individuals. One study examined the expression of SIRT1 and antioxidant enzymes (superoxide dismutase and catalase) in patients with heart failure with preserved ejection fraction before and after a four-week aerobic training program on a cycle ergometer at 50% of VO_2_max. The intervention increased SIRT1 expression in PBMCs and elevated serum antioxidant enzymes, supporting a cardioprotective role for SIRT1 in conjunction with enhanced oxidative defense. A subsequent study by the same group confirmed these results and further demonstrated that the training protocol elevated β-hydroxybutyrate (β-OHB) and antioxidant capacity (assessed via the TEAC assay), while lowering oxidized low-density lipoprotein. Linear regression analyses revealed that increases in β-OHB and SIRT1 were associated with reduced oxidative stress (Corbi et al. 2019).

Another study investigated the effects of moderate-intensity systematic balance training on SIRT1 concentrations in healthy older adults and individuals with Parkinson’s disease. In healthy participants, serum concentrations of both SIRT1 and SIRT3 increased significantly after training, whereas in the Parkinson’s disease group only SIRT1 showed a significant increase (Juan et al. 2023). Moreover, accumulating evidence indicates that individuals with a lifelong history of structured training, such as master athletes, exhibit higher SIRT1 concentrations compared with untrained middle-aged individuals (Aguiar et al. 2022; Koltai et al. 2018; Leite et al. 2024, 2023).

**Fig. 4:**
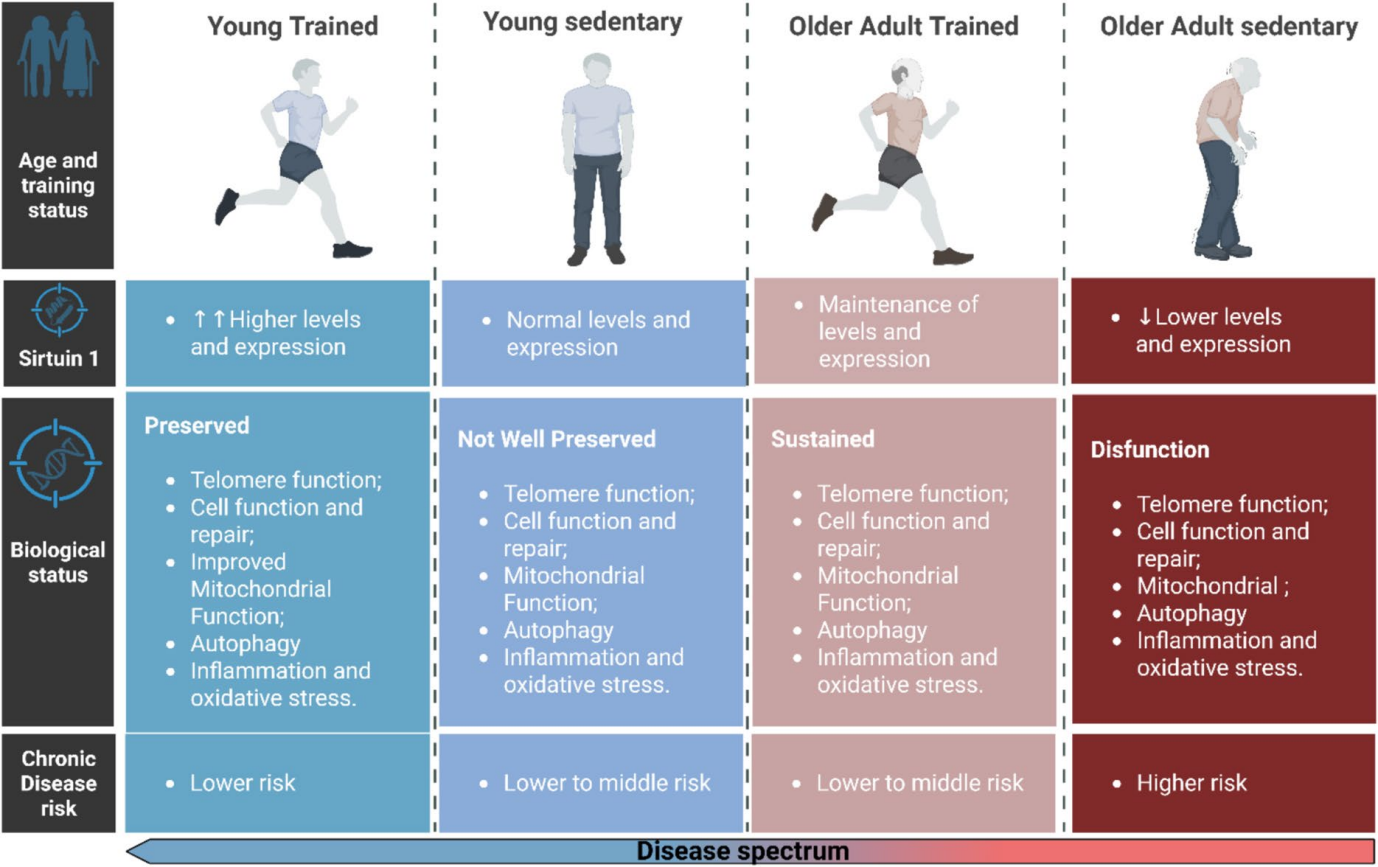
Main effects of SIRT1 induced by exercise training in sedentary/trained or young/older adults. Exercise training induces higher levels of SIRT1 that improves mitochondrial function, autophagy and cell repair, thereby reducing the risk of chronic diseases in older adults. On the one hand, a sedentary lifestyle promotes telomere dysfunction, inflammation, and oxidative stress independent of age. Biorender® web-based software was used to create the figure

Figure 4 illustrates the relationship among age, training status, SIRT1 levels, and chronic disease risk in young and older adults.

### Sex-specific adaptations in SIRT1 during aging and exercise

Research on the influence of sex on SIRT1 during aging remains in its early stages; however, some studies have begun to elucidate potential sex-specific effects on SIRT1 activity. One elegant study examining the effects of aging on SIRT1 activity in men and women reported that only women exhibited a significant decline in SIRT1 activity with advancing age, following a peak around 30 years. Moreover, in women, SIRT1 activity was positively correlated with triglyceride levels and basal metabolic rate, indicating that serum SIRT1 may follow sex-dependent patterns (Lee and Yang 2017).

Interestingly, downregulation of SIRT1 has been observed in the hearts of older women. This reduction was accompanied by diminished mitochondrial antioxidant defense and a pro-inflammatory shift, which were not present in the hearts of older men (Barcena de Arellano et al. 2019). In contrast, certain SIRT1 single-nucleotide polymorphisms have been associated with reduced mortality risk in older men, underscoring a sex-dependent protective effect (Ji et al. 2022).

Regarding exercise, a single bout of sprint interval training increased SIRT1 levels post-exercise, with no differences observed between men and women (Dun et al. 2024). However, emerging evidence suggests that chronic exercise may promote sex-specific adaptations in SIRT1. A six-month high-intensity aerobic training program upregulated SIRT1 expression in the vastus lateralis of adults over 50 years of age, with women showing higher levels than men (Ryan and Li 2023). Despite these findings, little is known about how SIRT1 responds to physical exercise across different metabolic tissues in females versus males of varying ages. Elucidating these sex-based differences across the lifespan is essential for developing tailored exercise interventions and optimizing health outcomes.

## Current SIRT1 challenges and perspectives for all physiological systems

SIRT1 overexpression or silencing are widely used techniques in cell culture to investigate gene function and develop potential therapeutic strategies for cellular senescence, thereby providing insights into age-related diseases and therapeutic targets (Huang et al. 2008; L. Zhu et al. 2014). More recently, organoids and spheroids, three-dimensional cell culture models, have emerged as advanced platforms for studying age-associated changes in tissues and organs with greater accuracy than traditional two-dimensional methods (Hu et al. 2018). Organoids, with their complex architecture and multiple cell types, offer sophisticated models for investigating tissue-specific aging. Spheroids, although structurally simpler, are also valuable, particularly when integrated into microphysiological systems (organ-on-a-chip) to study systemic aging and inter-organ communication (Živković and Opačak-Bernardi 2025).

Although several studies have elegantly investigated molecular mechanisms using SIRT1 tissue-specific knockout mice (Chen et al. 2008; Sanz et al. 2019), few have examined aged knockout rodents subjected to exercise training (Myers et al. 2019; Zhu et al.; 2023). This approach directly links gene function to cellular and organ-level effects, facilitating advances in target discovery, disease modeling, and the characterization of complex aging phenotypes. Recently, animal models of Alzheimer’s disease and Down syndrome have gained increasing attention for their ability to shed light on mechanisms underlying accelerated aging (Chen et al. 2008; Myers et al. 2019; Sanz et al. 2019). These models may pave the way for future therapeutic strategies that exploit the regulatory role of SIRT1.

The interplay between SIRT1-mediated deacetylation and phosphorylation in regulating other key proteins in response to exercise requires more in-depth investigation to clarify how exercise improves mitochondrial function. To better uncover exercise-induced changes in aged tissues, it is essential to apply integrated omics analyses that combine genomics, transcriptomics, and proteomics with state-of-the-art methods such as single-cell omics and spatial sequencing. These molecular profiling strategies can provide a comprehensive view of aging biology, identify central regulatory nodes and signal pathways, and reveal novel biomarkers, ultimately advancing precision therapies to promote healthy aging (Basilicata et al. 2025).

Current research on the impact of aging and physical activity on mitochondrial structure has largely focused on skeletal muscle, often neglecting other important metabolic tissues. High-resolution transmission electron microscopy remains a powerful technique for detailed morphological analysis and should be applied more broadly. Future studies should investigate how SIRT1 shapes specific mitochondrial features and examine energy flow and mitochondrial architecture in conjunction with other organelles, as this integrated perspective may reveal novel insights into the complex networks operating within and between cellular compartments.

The specific mechanisms and pathways involved in SIRT1 degradation during cellular senescence and exhaustive exercise, including its interaction with the autophagy–lysosome system, require further detailed investigation. Potential interactions between other mitochondrial sirtuins (SIRT3–SIRT6) and SIRT1 in the context of aging also warrant additional study. While genetic variants in the SIRT1 gene have been associated with longevity, more research is needed to clarify how these genetic differences influence SIRT1 expression and function during exercise. Furthermore, the role of SIRT1 in different cellular compartments, such as the cytosol, mitochondria, and nucleus, during chronic exercise in aged organisms has not yet been clearly established.

In human research, some studies have been conducted with heterogeneous, relatively small samples, and their core findings require validation in larger cohorts of older adults. Moreover, classifying participants as “high” or “low” responders, where even slight differences may have meaningful clinical effects, could help explain key training adaptations. The precise dose–response relationship between individual SIRT1 expression levels and clinical outcomes has yet to be determined. Additionally, SIRT1 function in older adults has been studied primarily in skeletal muscle and blood circulation, while other human tissues such as adipose tissue, heart, and brain remain challenging to investigate.

Further research is needed to determine how variations in exercise intensity, frequency, and duration affect SIRT1 profiles in each sex. Direct comparisons of aerobic training, resistance training, and combined protocols are particularly important to clarify how each modality influences SIRT1 signaling pathways. Moreover, internal and external factors regulating SIRT1 expression in adults over 80 years of age remain largely unexplored. Results from these studies could help health professionals design interventions to preserve and enhance health during aging. Identifying the main determinants of SIRT1 activity is clinically relevant and may guide the development of more effective rehabilitation programs.

**Fig. 5:**
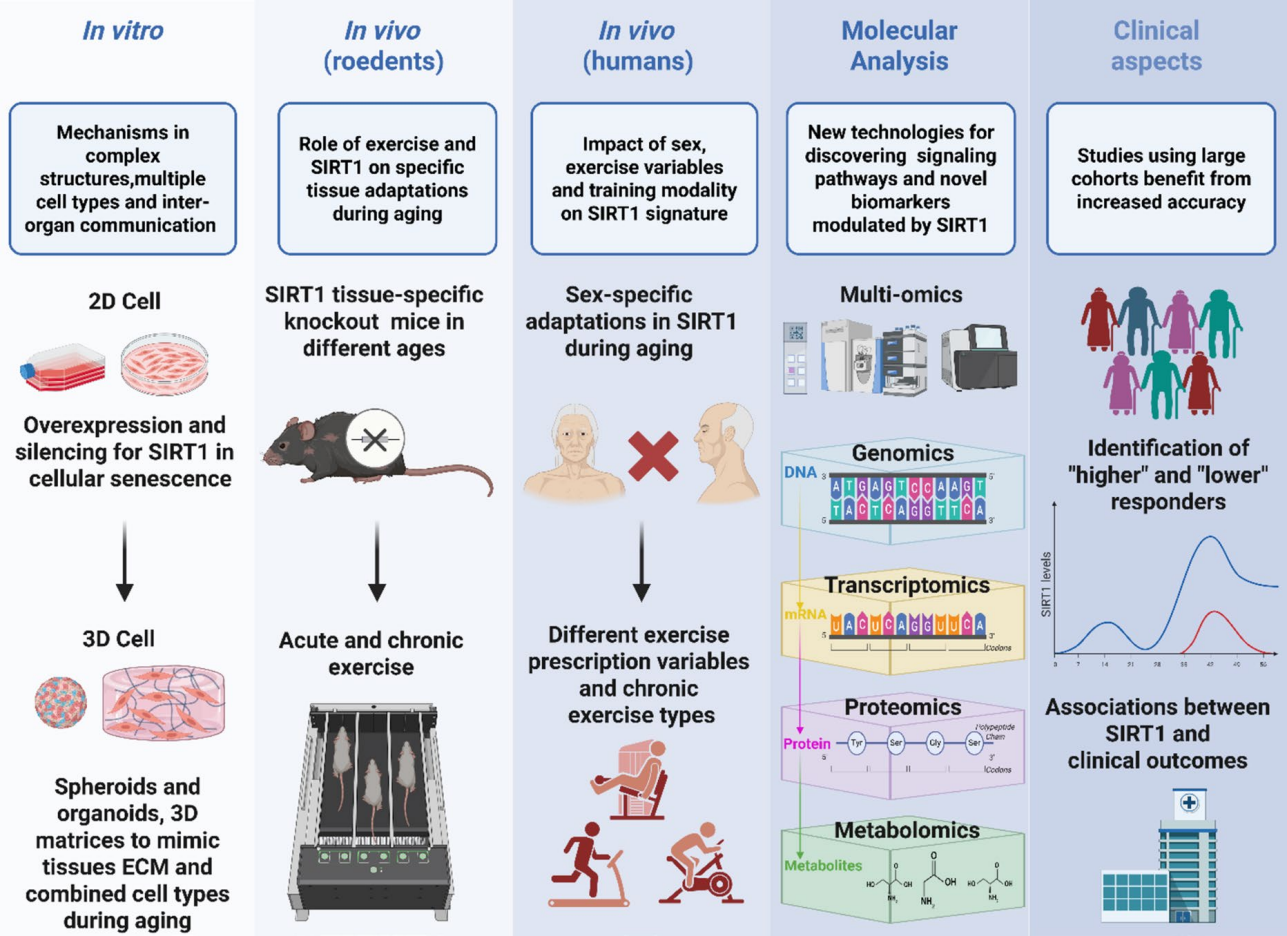
Basic and applied research on SIRT1 in aging: From contemporary investigations to future studies. In vitro approaches have investigated silencing or overexpressing SIRT1 in cellular senescence, but spheroids and organoids can offer new opportunities to study SIRT1 mechanisms across multiple cell and organ communication pathways. Also, the studies focused on aged rodents. However, SIRT1 tissue-specific knockout mice during different ages exposed to exercise must be established. Further research into how variations in exercise type and variables affect SIRT1 signature in each sex is needed to clarify how each method influences SIRT1 signaling pathways. The investigations focused on multi-omics techniques to discover new mechanisms and targets modulated by SIRT1. Finally, studies using large cohorts that establish an association between SIRT1 response and improvement in clinical outcomes are relevant. Biorender® web-based software was used to create the figure

Figure 5 summarizes current findings and outlines future translational directions for basic and applied research on SIRT1.

## Conclusion

In summary, growing evidence has demonstrated the pivotal role of SIRT1 in aging and its association with a wide range of age-related diseases and biomarkers. Optimal SIRT1 levels contribute to the maintenance of cellular homeostasis and the biochemical environment required for longevity by regulating mitochondrial dynamics, metabolic pathways, tissue remodeling, autophagy, inflammatory responses, and redox balance. These findings suggest that SIRT1, as a pleiotropic molecule, elicits multifaceted responses throughout the aging process.

SIRT1 also functions as a dynamic sensor of exercise-induced benefits and serves as a guardian of genomic integrity, thereby protecting against age-related decline. Distinct exercise protocols (acute and chronic) and modalities (aerobic, resistance, and combined training) have been shown to upregulate SIRT1 mRNA expression, activity, or protein levels across multiple tissues and organs in aged animals and older adults, thereby supporting health maintenance. Collectively, these observations support the proposal that SIRT1 is a potential exerkine and highlight that recognizing its role in exercise-induced adaptations offers new insights into non-pharmacological strategies to counteract the deleterious effects of aging.

## Data Availability

No datasets were generated or analysed during the current study.
